# Novel Highly Luminescent Amine-Functionalized Bridged Silsesquioxanes

**DOI:** 10.3389/fchem.2017.00131

**Published:** 2018-01-15

**Authors:** Rui F. P. Pereira, Sílvia C. Nunes, Guillaume Toquer, Marita A. Cardoso, Artur J. M. Valente, Marta C. Ferro, Maria M. Silva, Luís D. Carlos, Rute A. S. Ferreira, Verónica de Zea Bermudez

**Affiliations:** ^1^Chemistry Center, University of Minho, Braga, Portugal; ^2^Chemistry Department and CICS – Health Sciences Research Centre, University of Beira Interior, Covilhã, Portugal; ^3^Institut de Chimie Séparative de Marcoule - UMR 5257, CEA, CNRS, ENSCM, Université de Montpellier, Marcoule, France; ^4^Chemistry Department and CQ-VR, University of Trás-os-Montes e Alto Douro, Vila Real, Portugal; ^5^Chemistry Department, University of Coimbra, Coimbra, Portugal; ^6^Materials and Engineering Department and CICECO - Aveiro Institute of Materials, University of Aveiro, Aveiro, Portugal; ^7^Department of Physics, CICECO - Aveiro Institute of Materials, University of Aveiro, Aveiro, Portugal

**Keywords:** bis[(3-trimethoxysilyl)propyl]amine, sol-gel chemistry, solvent-assisted structuring, morphology, luminescence

## Abstract

Amine-functionalized bridged silsesquioxanes (BSs) were synthesized from bis[(3-trimethoxysilyl)propyl] amine via a solvent-mediated route. BS-1 and BS-2 were obtained at neutral pH with sub- and stoichiometric amounts of water, respectively, and high tetrahydrofuran content. BS-3 was prepared with hyperstoichiometric water concentration, high tetrahydrofuran content, and hydrochloric acid. BS-4 was synthesized with hyperstoichiometric water concentration, high ethanol content, and sodium hydroxide. BS-1 and BS-2 were produced as transparent films, whereas BS-3 and BS-4 formed white powders. Face-to-face stacking of flat or folded lamellae yielded quasi-hydrophobic platelets with emission quantum yields of 0.05 ± 0.01 (BS-1 and BS-2) or superhydrophilic onion-like nanoparticles with exciting emission quantum yields of 0.38 ± 0.03 (BS-3) and 0.33 ± 0.04 (BS-4), respectively. The latter two values are the largest ever reported for amine-functionalized siloxane-based hybrids lacking aromatic groups. Fast Grotthus proton hopping between = ${\text{NH}}_{2}^{+}$/ = NH groups (BS-3) and = N^−^/ = NH groups (BS-4), promoted by H^+^ and OH^−^ ions, respectively, and aided by short amine-amine contacts provided by the onion-like morphology, account for this unique optical behavior.

## Introduction

Bridged and non-bridged organosilanes [(RO)_3_-Si–R'–Si(OR)_3_ and R'–Si(OR)_3_, respectively] are key precursors for the development of bridged silsesquioxanes (BSs) and non-bridged silsesquioxanes (NBSs), respectively. BSs are an outstanding class of hybrid materials exhibiting a myriad of compositions, properties, and functionalities, whose synthesis relies on the versatile and rich organosilicon chemistry (Loy and Shea, 1995). In addition, BSs exhibit some important advantages, in particular the high organic loading possible under preservation of the mechanical stability. If prepared by the sol-gel process solely, BSs are obtained as transparent amorphous xerogels. Ordered BSs with hierarchical nanostructures and well-defined morphologies at the macroscopic scale may be produced, however, if the sol-gel method is combined with self-assembly routes (Loy and Shea, 1995; Moreau et al., 2001; Lerouge et al., 2006; Shimojima and Kuroda, 2006). In the latter case, two approaches are possible: structuring may be achieved either in the absence or in the presence of structure directing agents (self-directed assembly or template-assisted self-assembly, respectively).

In self-directed assembly the driving forces are weak interactions (van der Waals and/or π-π interactions and/or hydrogen bonding) (Boury and Corriu, 2002). BSs incorporating hydrophobic alkyl chains and suitable cross-links composed of hydrogen donor and acceptor groups able to establish hydrogen bonding interactions (e.g., a urea or an amide groups) have been extensively explored (Moreau et al., 2001; Besson et al., 2010; Fernandes et al., 2011b; Nunes et al., 2015b). A convenient and widely used strategy to promote structuring in NBSs is the use of non-bridged organosilanes carrying exclusively pendant alkyl chains (Parikh et al., 1997; Shimojima et al., 1997) or alternatively alkyl chains and suitable cross-links (Carlos et al., 2007; Nunes et al., 2012, 2013, 2014a,b, 2015a; Fernandes et al., 2013). A completely different approach was introduced by Besnard et al. (2013, 2016) to tune the structure of aminoundecyltriethoxysilane-derived silsesquioxanes in acidified water. In this case bending of the aliphatic chains/water interface was induced by ammonium headgroups through proton transfer from the acid to the amine group. Using the acid as curvature agent, these authors were able to fine tailoring the organization of the material from bilayers, multibilayer vesicles, cylindric direct micelles to nanofibers upon increasing the size of the acid. Very recently the judicious design of click chemistry-derived bridged organosilane precursors incorporating an amine group and a triazole ring comprising on a single position pendant alkyl chains with variable length, plus the control of acid concentration, allowed to tune the structure of the as-derived BSs from lamellar bilayer to a mixture of lamellar bilayer and a hexagonal 2D phase (Nunes et al., 2017). In this system the curvature agent responsible for the formation of the latter structure, scarcely reported in the literature, was the chloride ion counterbalancing the charge of the proton located between the amine group and the N(2) atom of the triazole ring.

**Figure 1:**
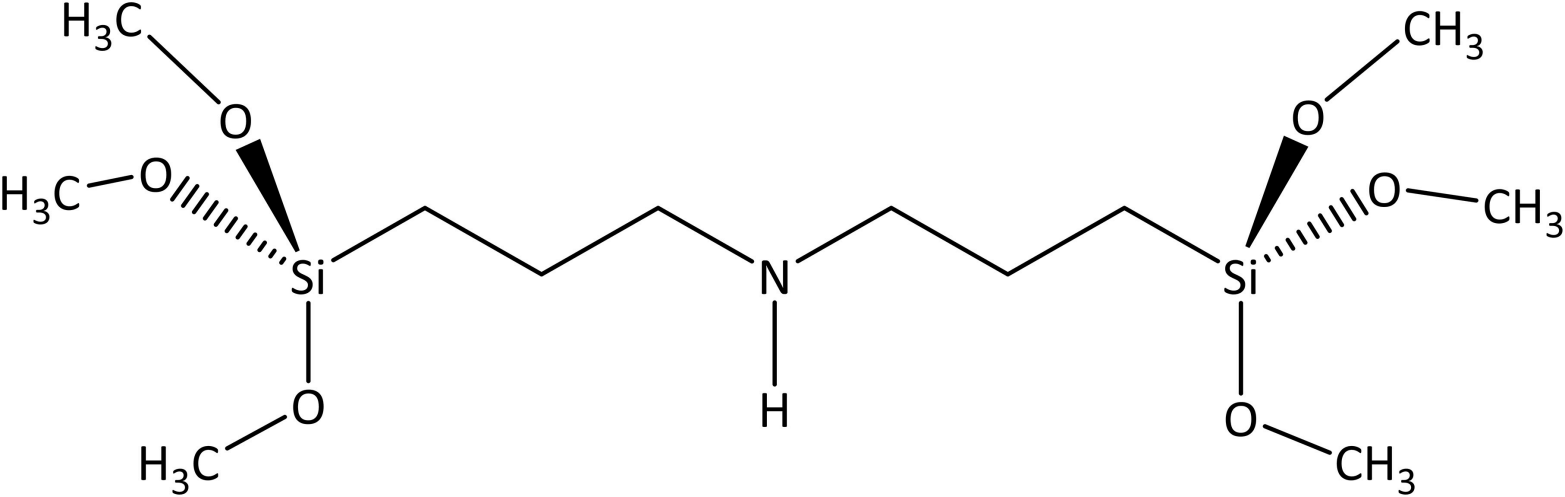
Structure of the BTMSPA precursor.

The existence of amine functionalities in ordered hybrid materials is quite attractive. For instance, amine-based periodic mesoporous organosilica (PMO) materials have foreseen applications in a wide variety of areas, ranging from base catalysis (Macquarrie and Jackson, 1997; Kantam et al., 2000; Demicheli et al., 2001; Huh et al., 2004; Wang et al., 2005), coupling and immobilization of functional molecules and biomolecules (Han et al., 1999; Lei et al., 2002; Chong and Zhao, 2004), and drug delivery (Muñoz et al., 2003) to chemosensing (Descalzo et al., 2002), among others. Several PMO systems incorporating amine groups were produced as powders (Hossain and Mercier, 2002; Zhu et al., 2002; Wahab et al., 2003; Ojo et al., 2012) or films (Park et al., 2010) using different reaction conditions, classical structure directing agents (Zhu et al., 2002; Ojo et al., 2012; Velikova et al., 2013), and precursors [typically bis[(3-trimethoxysilyl)propyl] amine (BTMSPA), Figure 1].

Targeting the production of ordered amine-functionalized BSs with tunable physical-chemical properties, in the present work BTMSPA was subject to a combination of sol-gel reactions and self-directed assembly routes mediated by a judicious mixture of solvents. BTMSPA was selected because it is a particularly interesting bridged organosilane precursor. This dipodal coupling agent has great technological potential in many areas, including dentistry (Lung et al., 2012) and corrosion protection of metals (Zhu and Van Ooij, 2004). In the present context it was chosen, not only to guarantee a high concentration of amine functionalities in the final materials, but also on account of its short organic bridges which are convenient to impart rigidity to the final network. Owing to the presence of a secondary amine group in the organic spacer, BTMSPA has basic character (pKa ≈ 10) (Ojo et al., 2012). In terms of sol-gel reactivity, BTMSPA exhibits an unusually high hydrolysis rate in basic conditions due to localized clustering of solvents with the amine in the bridge, which lowers the activation energy for hydrolysis (Tan and Rankin, 2006).

Several reasons induced us to adopt a solvent-assisted synthetic procedure. In the last few years it has been recognized that solvent molecules may be extremely useful for the rational design and construction of advanced crystalline materials. It has been established that solvent templates may affect the assembly of coordination supramolecular systems in two different ways (Li and Du, 2011): (1) They may act as ligands and/or guests and remain in the crystalline lattice affecting the product structure; (2) They may act as structure-directing agents influencing the crystal growth, framework, and final morphology although they will not belong to the final structure. Solvent templates may be shape- and site-selective, depending on their polarity, dielectric constant, bulkiness, and interactions with the organic functional groups of the growing supramolecular molecule. There are already numerous successful examples of the use of solvents as templates in various domains of materials chemistry. For instance, a solvent-template method was used in the fabrication of electrolyte membranes with ion transport channels at the nanoscale level with foreseen application in vanadium flow batteries (Zhang et al., 2014). More recently, the cyclic ethers 1,4-dioxane and tetrahydrofuran (THF) were used as templates to direct the formation of porous metal–organic frameworks (MOFs) (Ding et al., 2015). A double-solvent mediated overgrowth method was developed by Chou et al. (2015) to create hierarchical porosity in the zeolitic imidazolate framework-8 (ZIF-8) via overgrowing a ZIF-8 shell in methanol solution on a ZIF-8 core with water adsorbed in the pores. Interestingly, Moreau et al. employed a controlled hydrolytic route (large excess of water in the presence of an acid catalyst) to produce an unprecedented self-organized hybrid silica exhibiting long-range lamellar structure (Moreau et al., 2001). The final morphology of this lamellar BS was changed from rigid platelets to micro-objects mimicking sea sponges upon simple mixing of the precursor with dimethylsulfoxide (DMSO) (Fernandes et al., 2011a). More recently tailoring of the structure, morphology and photoluminescence features of di-amide crossed-linked decyl/siloxanes (di-amidosils), produced by sol-gel chemistry and self-assembly routes from the same precursor, was achieved through a fine control of various reaction parameters (Nunes et al., 2015b).

The synthesis of the BSs produced here relied on the addition of optimized amounts of binary or ternary mixtures of three solvents extensively used in sol-gel chemistry (water, ethanol, and THF) in the absence of a catalyst or in the presence of a base or an acid. The structure, morphology, surface wettability behavior, and luminescence features of the resulting materials were characterized.

## Results and discussion

### Characterization of BSs hybrids

**Figure 2:**
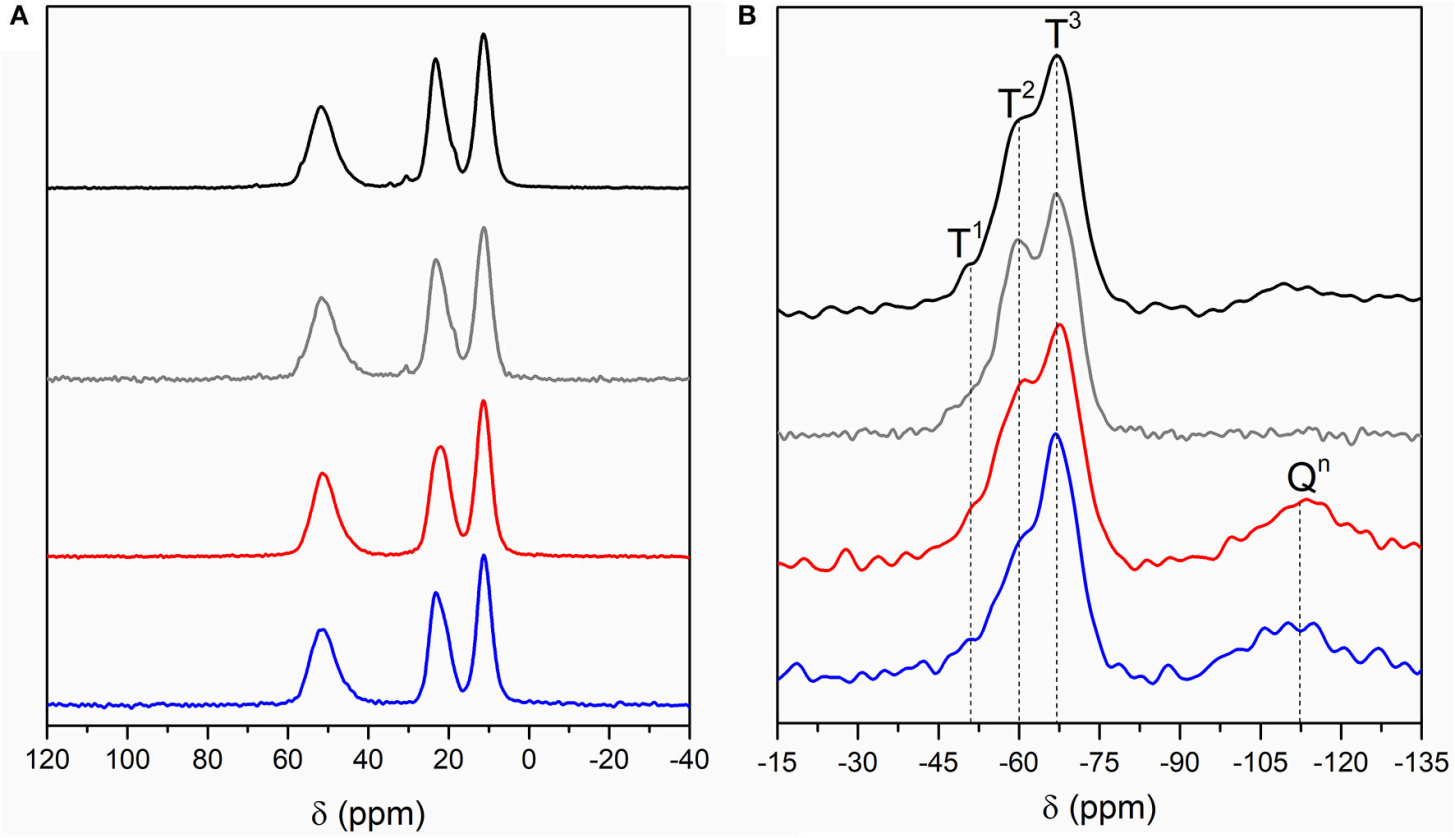
^13^C CP/MAS **(A)** and ^29^Si MAS **(B)** NMR spectra of the BS-1 (black line), BS-2 (gray line), BS-3 (red line), and BS-4 (blue line) hybrids.

Four BSs hybrid samples were synthesized (Supplementary Figure 1) and relevant details of the synthesis are given in Supplementary Table 1. The ^13^C CP/MAS and ^29^Si MAS NMR spectra of the BS-1, BS-2, BS-3, and BS-4 hybrids are reproduced in Figures 2A,B, respectively, and assigned in Table 1 (Park et al., 2010).

**Table 1:** ^13^C CP/MAS and ^29^Si MAS NMR data (δ in ppm) of the BSs hybrids. R' stands for –(CH_2_)_3_NH(CH_2_)_3−_.

**Sample**				**Attribution**
**BS-1**	**BS-2**	**BS-3**	**BS-4**	
**^13^C CP/MAS**				
51.7	51.7	51.5	51.6	SiCH_2_CH_2_***C***H_2_NH***C***H_2_
23.5	23.1	23.2	23.2	SiCH_2_***C***H_2_
11.3	11.2	11.3	11.2	Si***C***H_2_
**^29^Si MAS**				
−53.0 (2.7)	−53.4 (13.5)	−51.5 (8.8)	−50.7 (5.1)	T^1^ (%)
−59.6 (31.1)	−59.6 (30.9)	−59.8 (29.0)	−59.5 (23.1)	T^2^ (%)
−67.7 (51.6)	−67.6 (55.6)	−68.2 (38.7)	−67.4 (51.7)	T^3^ (%)
–	–	–	−87.8 (1.2)	Q^1^ (%)
–	–	−102.9 (5.4)	−99.6 (4.3)	Q^2^ (%)
–	–	−112.5 (11.1)	−109.7 (12.5)	Q^3^ (%)
–	–	−118.9 (7.0)	−117.0 (2.1)	Q^4^ (%)
84	81	83	88	*c*^a^
-	–	79	83	*d*^b^
R'_0.5_Si(O)_1.3_(OH)_0.5_	R'_0.5_Si(O)_1.2_(OH)_0.6_	–	–	Empirical formula

a *c = 1/3(%T^1^ + 2 × %T^2^ + 3 × %T^3^) × 100*.

b *d = 1/3(%T^1^ + 2 × %T^2^ + 3 × %T^3^) + 1/4(%Q^1^ + 2 × %Q^2^ + 3 × %Q^3^ + 4 × %Q^4^) × 100*.

The ^13^C CP/MAS spectra of the four BSs are practically identical (Figure 2A), demonstrating that the synthesis procedure did not affect the chemical structure of the materials. The absence in these spectra of the signal associated with the resonance of the methoxyl carbon atoms, expected at about 55 ppm, confirms that the hydrolysis of the Si-OCH_3_ groups was complete (Foston et al., 2013).

The dominant resonances of the ^29^Si MAS NMR spectra of the four BS samples (Figure 2B) are three signals detected in the −45 to −80 ppm interval ascribed to the following T^n^-type silicon sites of general formula (–CH_2_Si(OSi)_n_(OH)_3−n_) (Carlos et al., 2007; De Monredon–Senani et al., 2009): T^1^ (–CH_2_Si(OSi)(OH)_2_), T^2^ (–CH_2_Si(OSi)_2_(OH)), and T^3^ (–CH_2_Si(OSi)_3_) (Table 1). No significant T^0^ (CH_2_Si(OH)_3_) (Hook, 1996; De Monredon–Senani et al., 2009) environments associated with residual trihydroxysilane moieties could be detected in any of the four samples. In addition, the non-existence of the resonances characteristic of Q^n^-type environments (Si(OSi)_4−n_(OH)_n_) (Hook, 1996) in the ^29^Si MAS NMR spectra of the BS-1 and BS-2 hybrids confirmed that no cleavage of the Si–C bonds occurred during the synthesis. In the case of BS-3 and BS-4, the emergence of a broad peak around −110 ppm indicates the existence of a small proportion of Q^n^-type sites (ca. 20%), a result that suggests the non-preservation of the Si–C bonds in a fraction of these two hybrids. Okamoto et al. (2005) also reported the partial cleavage of the Si-C bond in the non-porous lamellar hybrid solids obtained from the base-catalyzed precursors 1,4-bis(triethoxysilyl)benzene (1,4-BTEB), 1,4-bis(triethoxysilyl)benzene (1,3-BTEB), and 4,4′bis(triethoxysilyl)biphenyl (4,4′-BTEBp). Based on the relative populations of the T^1^, T^2^, T^3^, and Q^n^ environments, polycondensation degrees *c* of 84 and 81% were calculated for BS-1 and BS-2, respectively, and polycondensation degrees *d* of 79 and 83% were calculated for BS-3 and BS-4 hybrids, respectively (Table 1). The magnitude of the *c* and *d* values points out the formation of a highly polycondensed 3D siloxane network in the four materials, the highest degree of ramification being attained in BS-4. The empirical formulae deduced for BS-1 and BS-2 demonstrate that some OH groups persisted bonded to the Si atoms.

**Figure 3:**
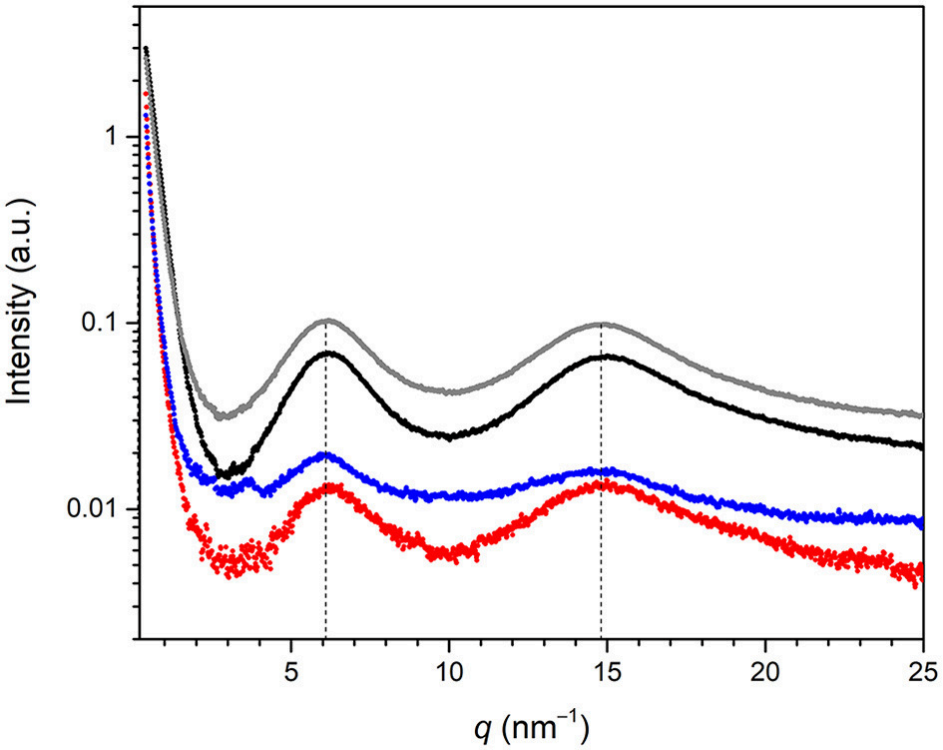
SWAXS spectra of the BS-1 (black line), BS-2 (gray line), BS-3 (red line), and BS-4 (blue line) hybrids.

The SWAXS spectra of BS-1, BS-2, BS-3, and BS-4 in the 0.4–25 nm^−1^ *q* (where *q* = 4π sinθ/λ, 2θ is the scattering angle and λ is the wavelength) range are represented in Figure 3. The four samples produced two distinct broad peaks at 6.1 and 14.8 nm^−1^ in the analyzed *q* range. In an attempt to explain the origin of the former peak, molecular modeling performed with the MM2 model of ChemDraw Ultra software (version 12.0.2.1076) was applied to the BTMSPA precursor. The length deduced for the Si(CH_2_)_3_NH(CH_2_)_3_Si spacer (*d*_Si−Si_) was 1.07 nm (*q*_Si−Si_ = 5.90 nm^−1^), which is of the same order of magnitude of the distance associated with the scattered signal observed at about 6.1 nm^−1^ in the SWAXS spectra. This scattered signal is ascribed to the first of the *n*th-order peaks of a lamellar structure with spacing *l*_1_ = 1.03 nm. The peak seen around 14.8 nm^−1^ is attributed to the coherent diffracting domains of the siliceous backbone (*d*_Si−O−Si_ = 0.424 nm) (Carlos et al., 1999). The absence of peaks at higher *q*-values may be interpreted as an indication that the structural order did not extend over a long range. We note that N_2_ adsorption/desorption data (not shown) demonstrated the non-porous nature of the four samples.

It is useful to mention at this stage that Ojo et al. (2012) obtained two mesoporous BSs (so-called samples 1 and 2) also from BTMSPA, but in the presence of sodium dodecyl sulfate (SDS) and dodecylamine (DDA). The XRD patterns of both materials also exhibited two characteristic reflections solely, demonstrating short-range ordering: a prominent broad reflection at 14.8 nm^−1^ (*d*_Si−O−Si_ = 0.424 nm) and a less intense peak, also broad, at 6.4 nm^−1^ (*d* = 0.981 nm) and 6.1 nm^−1^ (*d* = 1.03 nm), respectively. The production of a non-porous lamellar BS or a mesoporous BS from the very same precursor in the absence or presence of a template, respectively, is well-known. The first hierarchically structured PMO including meso- and molecular-scale periodicity was obtained by Inagaki et al. (2002) from 1,4-bis(triethoxysilyl)benzene (1,4-BTEB) and octadecyltrimethylammonium chloride (OTMAC). Later Okamoto et al. (2005) reported that the hydrolysis and condensation of 1,4-BTEB, 1,3-BTEB, and 4,4′bis(triethoxysilyl)biphenyl (4,4′-BTEBp) in basic or acidic media lacking the presence of a surfactant led to the formation of non-porous lamellar hybrid solids with very long-range order. These findings induced Okamoto et al. (2005) to conclude that a periodic structure with alternating arrangement of organic and silica layers is essential for achieving long-range order in hybrid materials even in the absence of surfactants. These authors also pointed out that in surfactant-directed mesoporous organosilicas the role of the surfactant is to enhance the structural periodicity.

As expected, the birefringence manifested in the POM images of BS-1, BS-2, BS-3, and BS-4 recorded between crossed polarizers (Supplementary Figure 2) indicates submicrometric anisotropy.

**Figure 4:**
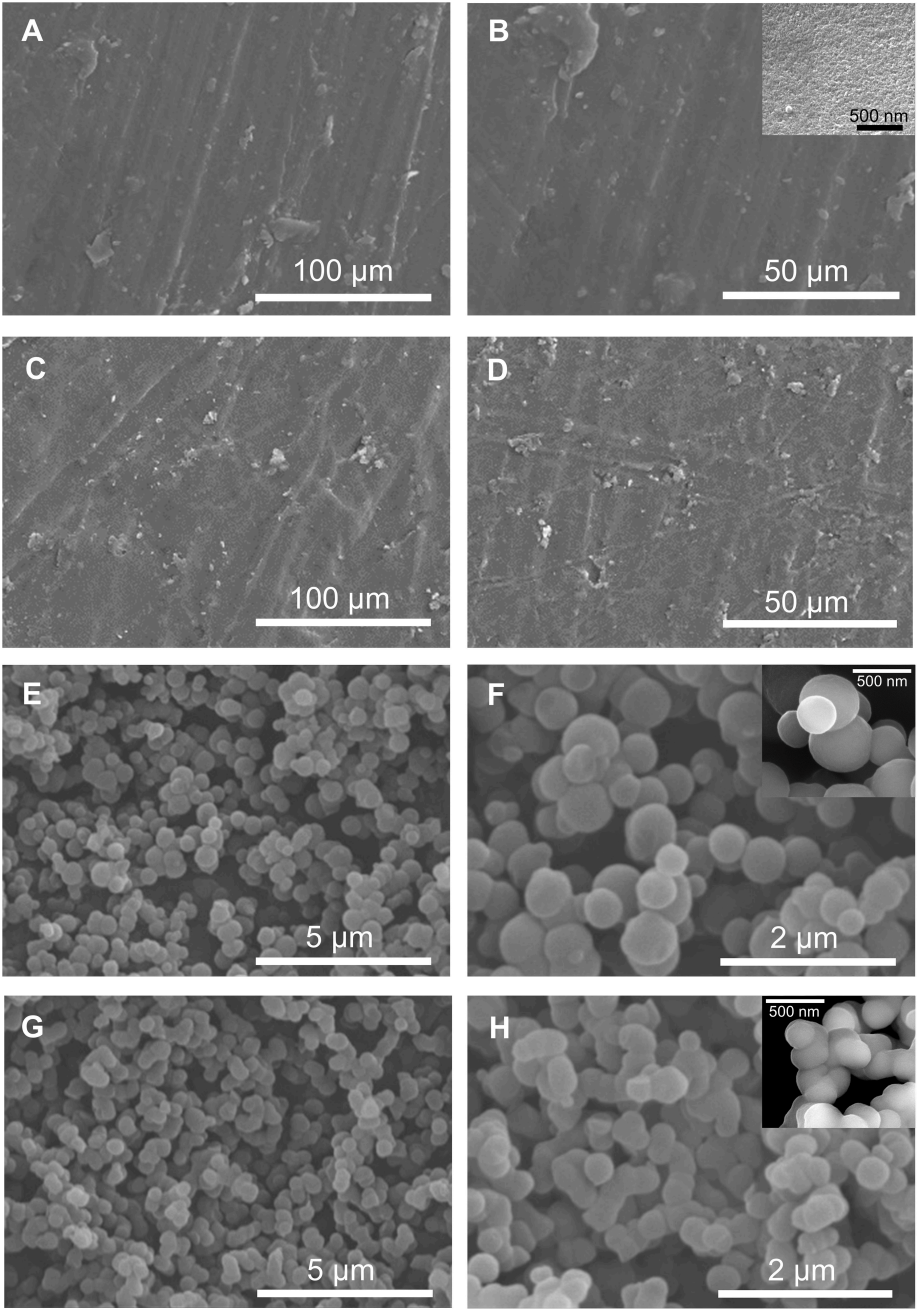
SEM images of the BS-1 **(A,B)**, BS-2 **(C,D)**, BS-3 **(E,F)**, and BS-4 **(G,H)** hybrids.

The SEM images reproduced in Figure 4 demonstrate the homogenous and irregular texture of BS-1 and BS-2. In contrast, the SEM images of BS-3 and BS-4 reveal the presence of interconnected compact and uniform nanosized spherical particles.

**Figure 5:**
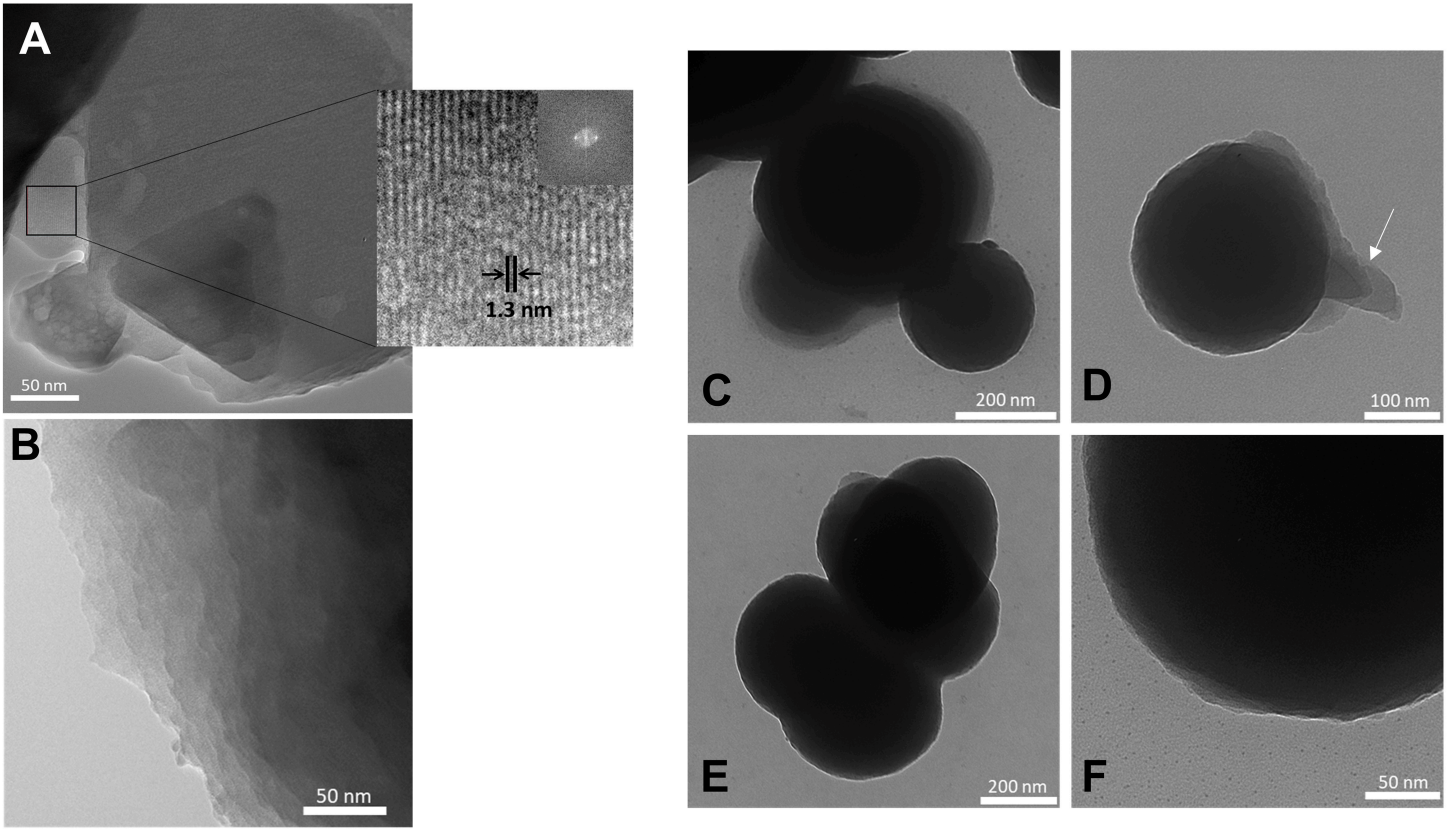
TEM images of the BS-1 **(A)**, BS-2 **(B)**, BS-3 **(C,D)**, and BS-4 **(E,F)** hybrids.

The TEM image of BS-1 (Figure 5A) shows areas with an ordered superposition of thin platelets (see right bottom area) coexisting with other areas with reduced order. The platelets exhibit parallel patterns with an average spacing of about 1.30 nm, thus of the same order of magnitude as the interlamellar distance retrieved from SWAXS (*l*_1_ = 1.03 nm). A different area of the same sample may be observed in the TEM image of Supplementary Figure 3. An organization along thin platelets is also clearly visible in the case of BS-2 (Figure 5B). The TEM image of Figure 5D, which depicts a single nanoparticle of BS-3, is particularly interesting. It reveals a series of thin platelets at the surface of this nano-object (see white arrow), therefore strongly suggesting that the formation of these nano-spheres may have occurred along an onion-like fashion. This sort of assembly process (also seen in Figures 5C,E,F) would explain the preservation of the lamellar structure of the platelets in BS-3 and also in BS-4, as indicated by SWAXS.

**Figure 6:**
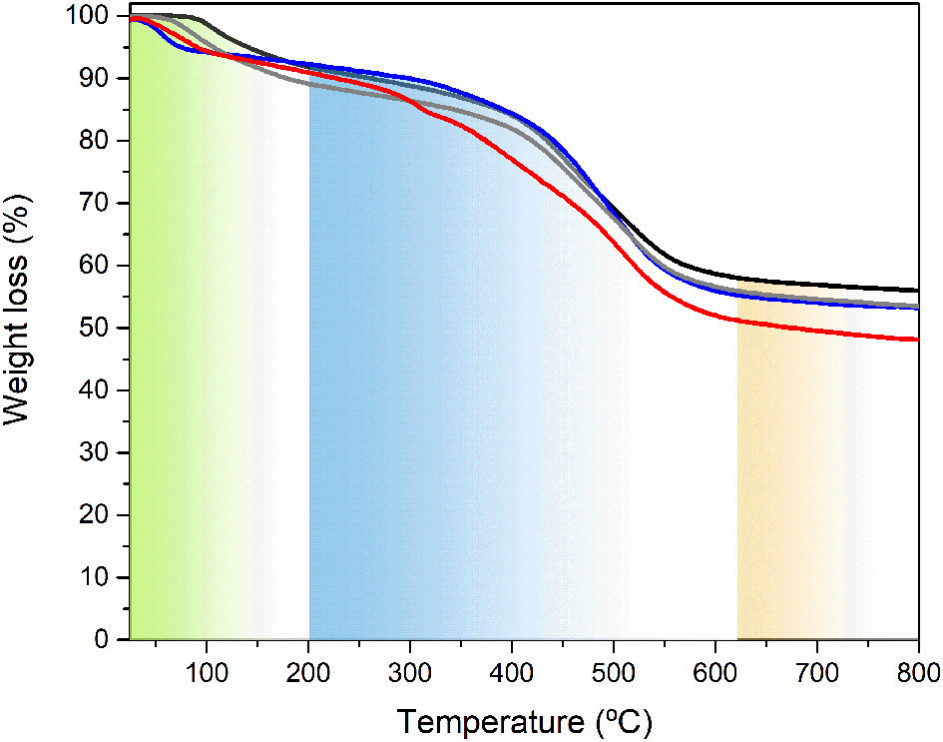
TGA curves of the BS-1 (black line), BS-2 (gray line), BS-3 (red line), and BS-4 (blue line) hybrids.

The TGA curves of the BS-1, BS-2, BS-3, and BS-4 hybrid samples in the 25–800°C temperature range are depicted in Figure 6. Based on the results of the DSC analysis (Supplementary Figure 4), the first weight loss observed is correlated with the removal of occluded solvent(s) present in the samples. According to these data, the content of the solvent(s) in these samples followed the trend: BS-1 < BS-2 < BS-3 < BS-4. For BS-1 and BS-2 the weight loss was significantly less dramatic and started at higher temperatures than in the case of the other two samples (BS-3 and BS-4). Moreover, the endset of solvent evaporation occurred at higher temperature for BS-1 and BS-2 (around 250°C) than for BS-3 and BS-4 (around 175°C). This stage was followed by a plateau, in which no significant variation of weight took place. The degradation of the organic spacer occurred via the cleavage of the C-C and C-N bonds of the propyl chains and was initiated earlier in the case of BS-3 and BS-4 with respect to the situation found for BS-1 and BS-2.

Beyond this stage, thermal decomposition was accompanied by a sharp mass loss up to ca. 650°C. Between the latter temperature and 800°C, the weight loss rate decreased markedly. At the highest temperature analyzed, approximately 42, 45, 50, and 45% of the BS-1, BS-2, BS-3, and BS-4 hybrids remained to be decomposed, respectively.

**Figure 7:**
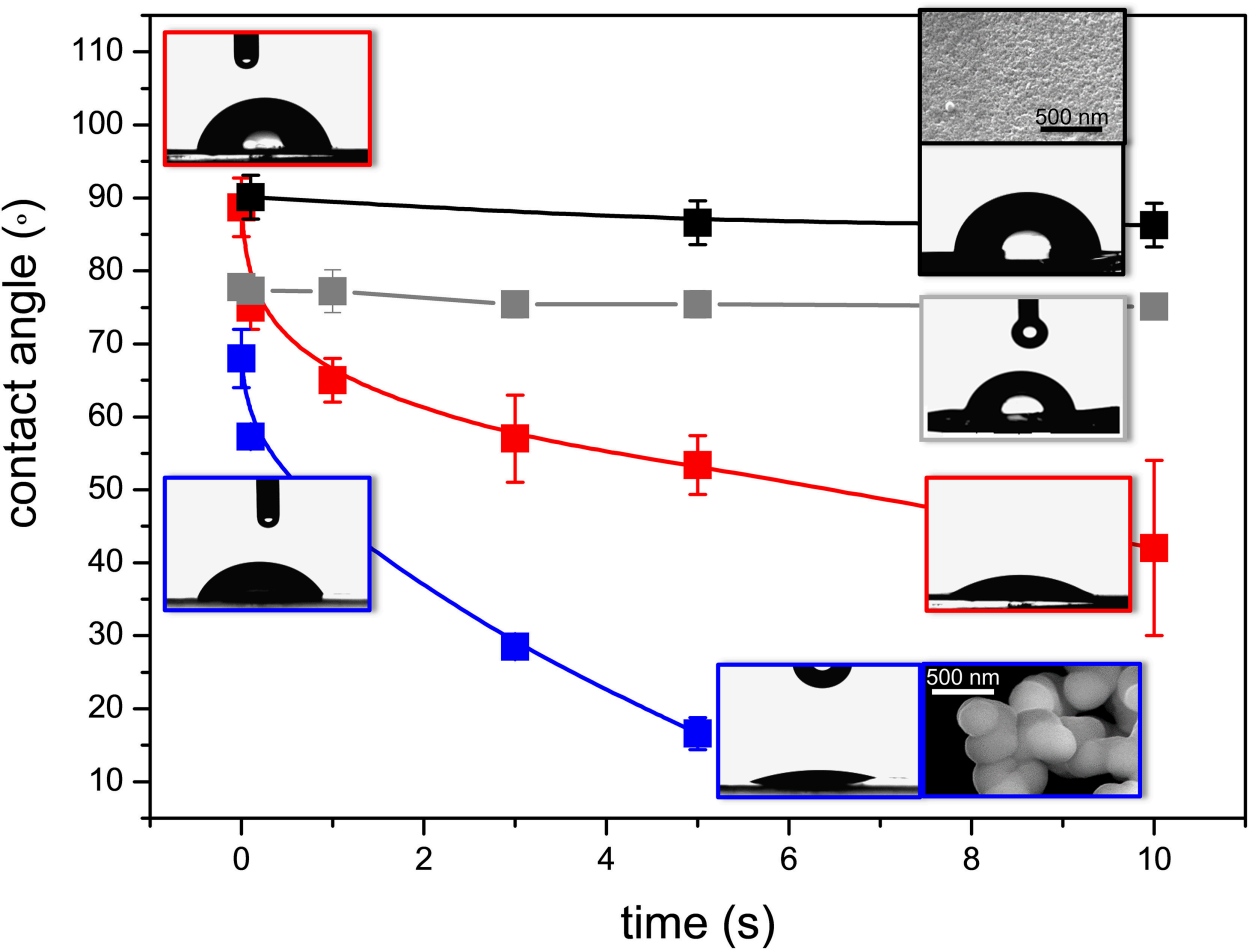
Time-dependence of the contact angle of the BS-1 (black line), BS-2 (gray line), BS-3 (red line), and BS-4 (blue line) hybrids. Insets: HR-SEM images of BS-1 and BS-4.

The variation of contact angle (θ) as a function of time for the prepared hybrids, represented in Figure 7, reveals the superhydrophilic nature of BS-3 and BS-4, with the corresponding θ-values undergoing a two- and four-fold decrease after 10 and 5 s, respectively (Supplementary Table 2). As expected, drop spreading was marked for both these samples (see corresponding images in the insets of Figure 7). In contrast, BS-1 and BS-2 displayed a considerably less hydrophilic behavior, with practically no change of θ within the same period of time. The initial value measured for BS-1 was 90° (Supplementary Table 2), a value that is considered as the ideal frontier between hydrophilic and hydrophobic behavior. In the case of BS-2, the value measured was slightly lower (78°).

### Mechanistic scenario

A mechanistic scenario will be proposed as follows to explain the significant differences detected both in the morphology and surface properties of the BS-1, BS-2, BS-3, and BS-4 materials derived from the same BTMSPA precursor.

The control of the structure of sol-gel derived materials is governed primarily by the kinetics of the sol-gel reactions of the precursor. It should be pointed out first that the rate of the sol-gel reactions always competes with the rate of the formation of the ordered mesophase and, when dealing with dilute solutions, precipitation (Cerveau et al., 2000; Boury and Corriu, 2002; Nakanishi and Kanamori, 2005; Soler-Illia and Innocenzi, 2006). The rate-controlling reactions of the sol-gel process (reversible hydrolysis and alcohol- or water-producing condensation) depend critically on the reaction conditions. In most cases in acidic media the condensation reactions are rate-limiting, whereas in basic media the hydrolysis is rate-limiting (Brinker and Scherer, 1990; Jiang et al., 2006). In the case of the production of ordered BS materials from bridged organosilane precursors incorporating hydrophobic organic spacers and appropriate cross-links (e.g., urea groups) the optimal synthesis conditions are well established. Structuring relies on the stabilization of amphiphilic organo(bis-silanetriol) structures mediated by the creation of a strong hydrogen-bonded network which then promotes ordering of the hydrophobic spacers (Cerveau et al., 2000). In this sort of BSs the formation of hydrogen bonding interactions between the silanol or other groups (e.g., urea cross-links) competes with the condensation reactions that yield the siloxane network. The creation of the ordered organo (bis-silanetriol) structure, aided by the hydrophobic interactions between the organic spacers, is favored under controlled hydrolysis (typically a large excess of water and the presence of an acid catalyst). Water exerts in this process an important role inducing the creation of a hydrophilic hydrogen-bonded network layer, while decelerating the formation of water via condensation reactions.

Also critical in structuring is the precursor reactivity, as it determines the range of conditions in which ordered materials can be obtained (Bao et al., 2006). Interestingly, while studying the influence of the structure of several non-bridged and bridged alkoxysilane precursors on sol-gel reactivity, Tan and Rankin (2006) reported that the behavior of BTMSPA in basic medium was atypical. These authors realized that the reactivity of bridged silanes toward hydrolysis in basic conditions could be explained in terms of inductive and steric factors. As the hydrolysis in basic medium is a nucleophilic substitution reaction, a high electron density at the silicon and bulky groups near a silicon site are expected to decelerate the reaction. Surprisingly the introduction of an amine group in the bridging chain of 1,6-bis(trimethoxysilyl) hexane (BTMSH) caused a large increase of the hydrolysis reaction rate difficult to explain. Intermolecular catalytic or electron inductive effects were immediately excluded. Furthermore, the acceleration was not consistent with the slightly larger bulkiness of the chain and the slightly electron donating nature of the amine. Based on *ab initio* calculations, which indicated that solvent cluster association with the reacting molecule might play a significant role in promoting sol-gel reactions (Kay and Assink, 1986; Assink and Kay, 1988), Tan and Rankin (2006) proposed that the acceleration effect detected in the case of BTMSPA was due to electrostatic and hydrogen bonding between the amine group in the bridging chain and water (or alcohol), which led to localized clustering of the solvent(s) near the silicon site. This solvent effect lowered the activation energy for hydrolysis, thus enhancing the hydrolysis rate.

Let us now focus on the four BTMSPA-derived samples obtained in the present work. The above SWAXS data provided evidence of the presence of a lamellar organization at the molecular scale in all the samples. However, the syntheses of BS-1 and BS-2, carried out in a large excess of THF, were rather slow processes that led to formation of free-standing transparent films composed of thin platelets, whereas the syntheses of BS-3 and BS-4, performed in the presence of a large excess of water, and a minor proportion of THF and ethanol, respectively, led rapidly to the formation of white powders composed of nanoparticles apparently built from an onion-like assembly of thin platelets. Furthermore, it was also observed that the hydrophilicity of BS-1 (θ ≈ 90°) was considerably lower than that of BS-4 (θ ≈ 10°). Clearly the radical differences found in the morphology and surface properties of the four samples can only be attributed to a solvent effect, probably acting in the late stages of the syntheses.

Before discussing in detail each situation, we recall that: (1) The production of BS-1 and BS-2 was carried out at pH = 8 in the presence of sub- and stoichiometric amounts of water, respectively, and using a significant amount of THF (molar ratios Si:H_2_O:CH_3_CH_2_OH:THF = 1:0.75:2:41.7 and Si:H_2_O:CH_3_CH_2_OH:THF = 1:1.5:4:41.2, respectively). The molar fractions of water (XH_2_O)/THF (X_THF_) in the mixtures were 0.017/0.938 and 0.032/0.882, respectively, indicating that the solvent mixtures contained virtually only THF; (2) To obtain BS-3 an amount of THF slightly lower than that employed for BS-1 and BS-2 was first incorporated, then a hyper-stoichiometric amount of water and a minor content of HCl were introduced (molar ratio Si:H_2_O:HCl:THF = 1:300:0.1:35; X_H2O_ = 0.896 and X_THF_ = 0.104). In this case, the solvent mixture was essentially composed of water and the pH was 9; (3) At last BS-4 was prepared at pH = 12 by first dissolving BTMSPA in approximately 10 times the amount of ethanol employed in the case of the syntheses of BS-1 and BS-2, and then adding about half of the amount of water used in the case of the preparation of BS-3 and NaOH (molar ratio Si:H_2_O:CH_3_CH_2_OH:NaOH = 1:157.5:19.5:0.115; XH_2_O = 0.890). Here, the solvent mixture included essentially water too.

It is also essential to refer at this stage some physical-chemical features of the three solvents which are relevant for the present discussion. Water and ethanol are protic polar solvents with dielectric constants (ε) of 80.1 (20°C) and 24.5 (25°C), respectively, and dipole moments (μ) of 1.85 and 1.69 D, respectively. THF is a non-protic polar solvent with ε = 7.50 and μ = 1.75 D. The van der Waals volume of water, ethanol and THF are 11.44, 31.94 and 46.12 cm^3^ mol^−1^ (Li and Du, 2011).

**Figure 8:**
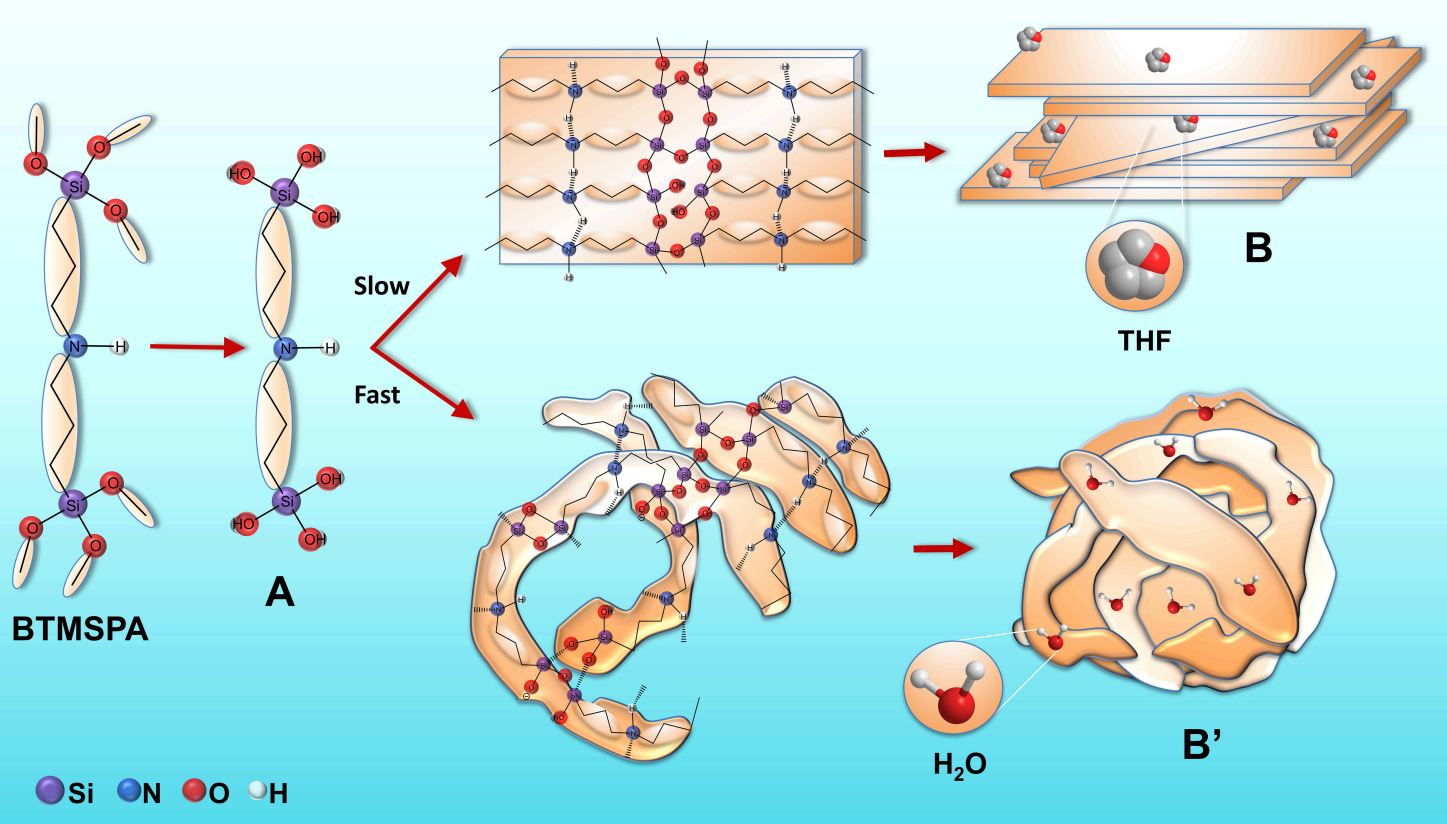
Mechanistic scenario for the synthesis of the BS-1 (top) and BS-4 (bottom) hybrids.

It is useful to examine first the two most extreme cases represented by samples BS-1 and BS-4. We propose that in the very early stages of the synthesis mechanism of BS-1 the addition of ethanol, water and THF to BTMSPA resulted in the formation of an organobis(silanetriol) compound (A, Figure 8). The formation of the siloxane network was induced by hydrogen bonding between the amine groups or between the silanol groups, and van der Waals interactions between the propyl chains, leading to the formation of thin, non-charged lamellar ribbons (B, Figure 8). Under the conditions employed (i.e., neutral pH and understoichiometric H_2_O/Si ratio for complete hydrolysis), the hydrolysis rate occurred at a minimum rate, the condensation reaction initially proceeded between incompletely hydrolyzed species and the depolymerization rate was relatively low. This implies that the initial structures, weakly branched, most likely resembled those obtained via reaction-limited cluster aggregation (RLCA) (Brinker, 1988). Water formed by condensation reactions favored more complete hydrolysis, increased the depolymerization rate and ultimately the extent of branching and restructuring. With the increase of the initial H_2_O/Si ratio, condensation and restructuring proceeded at the same time at more comparable rates and the growth process resembled then closely nucleation and growth (Brinker, 1988). The growing supramolecular architecture subsequently directed the ordering of the hybrid silica. Face-to-face stacking of the lamellar ribbons (B) yielded 3D thin platelets. We believe that the role of THF in the whole process was two-fold. Because it does not have available labile protons, this solvent is often considered in the sol-gel context as a “neutral” solvent, which acts essentially as a homogenizing agent, facilitating the hydrolysis of alkosysilanes (Brinker, 1988). Moreover, the hydrogen bonding interaction of THF with the silanol groups of the final material, leaving the THF hydrophobic rings at the surface, could account for the quasi-hydrophobic character of BS-1. Strong arguments supporting this explanation are the very significant concentration of silanol groups in this sample [as demonstrated by the low *c* value of the siloxane network (Table 1)] and the high solvent basicity (SB) (Catalán, 2001) of THF [(SB_THF_ = 0.591) (SB is an index related with the hydrogen-bond acceptor basicity of the solvent].

In the case of the synthesis mechanism of BS-4, the initial addition of ethanol to dissolve BTMSPA was crucial. This solvent, used as received and thus containing water, initiated the formation of the organobis(silanetriol) compound A (Figure 8). It is worth recalling that ethanol is also classed as a homogenizing agent (Brinker, 1988). The subsequent addition of a large excess of water and NaOH led to the immediate formation of bent ribbons (B', Figure 8) owing to the very fast hydrolysis. Indeed, in the presence of a basic catalyst and at high H_2_O:Si ratios, the hydrolysis of alkoxysilanes is considerably faster than condensation. As a consequence, highly branched and compact “colloidal” particle gels emerge (Brinker and Scherer, 1990). In addition, as already pointed out, BTMSPA exhibits an unusually fast hydrolysis rate in basic medium. The high pH of the reaction medium favored deprotonation of some silanol and amine groups of the ribbons and B' became negatively charged. Its stabilization was ensured by the high DC of the water/ethanol mixture which ensured high charge mobility and low charge repulsions. Face-to-face stacking of the folded lamellar ribbons B' (onion-like assembly) led ultimately to the formation of 3D nanoparticles. Stacking of these folded ribbons led to the progressive growth of nanoparticles. Hydrogen bonding of the water molecules to the siliceous network explains the high wettability of this sample.

The synthesis of BS-2 resembled closely that of BS-1 and, not surprisingly, the general properties of both samples were very similar. The conditions adopted to obtain BS-3 may be considered as an intermediate case between those used to produce BS-1 and BS-4. In fact the presence of THF in the reaction medium used to produce BS-3 induced the mechanism to shift slightly toward the conditions used in BS-1. This gives support to the increase of the θ value measured for BS-3 with respect to that of BS-4.

### Photoluminescence characterization

**Figure 9:**
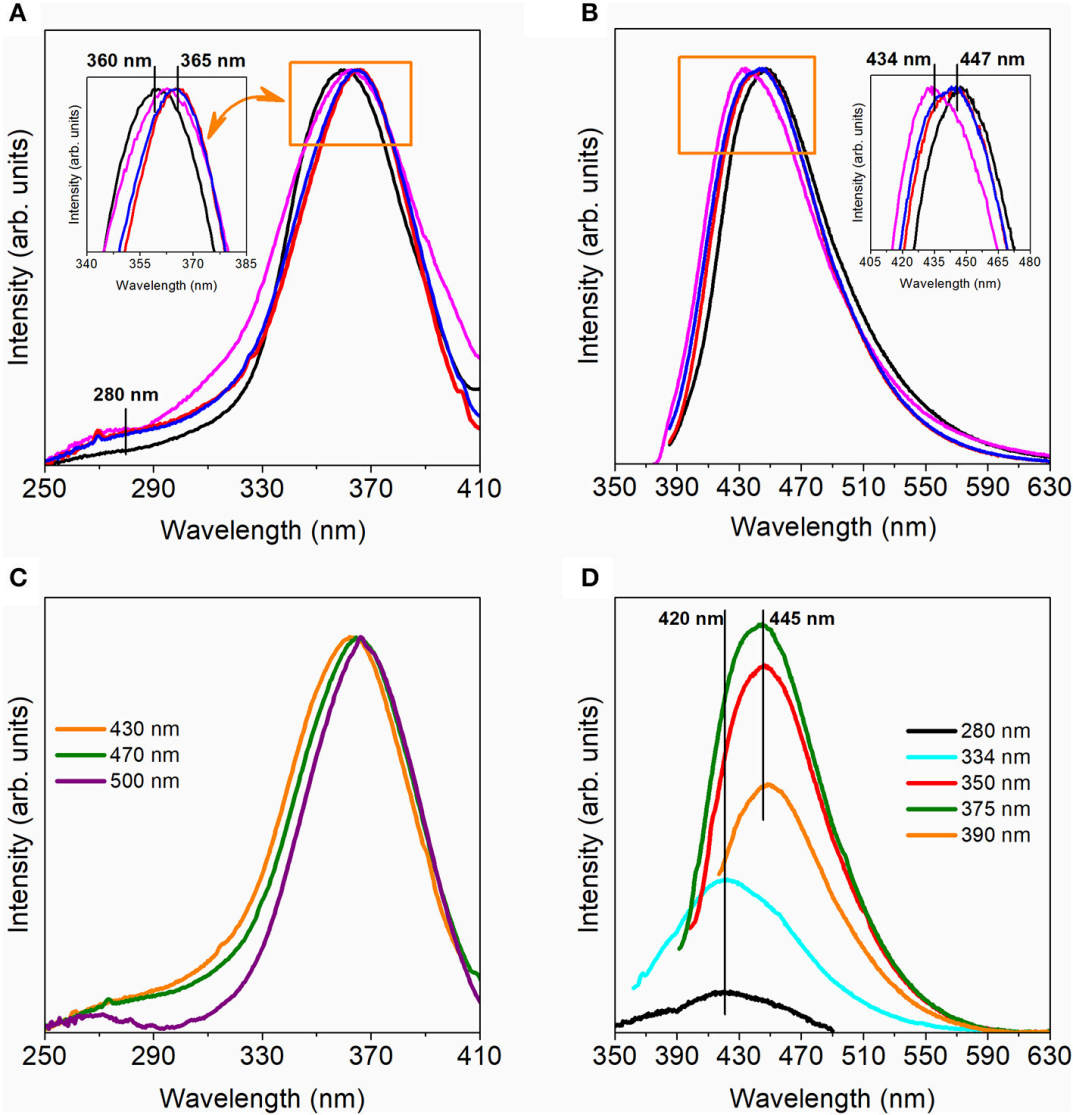
Excitation **(A)** and emission **(B)** spectra monitored around 445 nm and excited at 360 nm, respectively, of the BS-1 (black line), BS-2 (magenta line), BS-3 (red line), and BS-4 (blue line) hybrids. Excitation **(C)** and emission **(D)** spectra of BS-4 monitored and excited at distinct wavelengths.

Figure 9A shows the excitation spectra of the BS-1, BS-2, BS-3, and BS-4 hybrids monitored around 445 nm, revealing a main band in the ultraviolet (UV) spectral region whose peak energy slightly depends on the selected material, namely 360 nm for BS-1 and 365 nm for the remaining ones. A lower-relative intensity band around 280 nm is also discerned. These two components have been assigned to the siliceous- and NH-related excited states, respectively (Carlos et al., 2001, 2004). Under UV excitation, the hybrids displayed a broad band emission in the blue spectral region, whose peak energy is nearly independent of the material (Figure 9B). The excitation spectra dependence on the monitoring wavelength suggests the presence of localized states, as previously detailed (Ferreira et al., 2006a,b).

Comparison of these emission features with those of analogous amine-functionalized siloxane organic-inorganic hybrids leads us to conclude that such blue emission is originated by electron-hole recombinations occurring in the siliceous domains and amine groups (Carlos et al., 2001, 2004). Nevertheless, a distinct aspect is that in the case of the BS-1, BS-2, BS-3, and BS-4 hybrids the excitation and emission spectra are much less dependent of the monitoring and excitation wavelength, respectively, as illustrated in Figures 9C,D for a selected hybrid sample (see also Supplementary Figures 5–10). An analogous dependence of the emission spectra on the excitation wavelength was reported earlier for similar amine- or amide-functionalize hybrids and other amorphous semiconductors. The radiative transitions involving carriers excited into localized states were proposed as responsible for the excitation energy dependence of the emission energy in amorphous semiconductors (Ferreira et al., 2006a,b).

Focusing our attention on the excitation dependence of the emission (Figure 9D), we notice that at an excitation wavelength within the siliceous-related low-wavelength excitation component (280 nm), the emission is centered at 420 nm, whereas at the longer excitation wavelengths ascribed to the preferential excitation of the amine group-related states the emission peaks at 445 nm. The fact that both the siliceous- and NH-related components do not depend on the excitation wavelength suggests a strong decrease of the density of localized states within the energy band gap, preventing the electrons' thermalisation and the consequent emission red-shift. As the presence of localized states in the energy band gap is a strong evidence of disordered-related processes (e.g., amorphous semiconductors), we may suggest that BS-1, BS-2, BS-3, and BS-4 are characterized by a higher local order.

Another evidence that the local organization impacted on the photoluminescence features is inferred from the strong dependence of the emission quantum yield on the morphology of the samples which is in turn dictated by the synthesis route (type/concentration of solvent(s) and presence (or not)/type of catalyst). While in the case of the samples produced as films low emission quantum yield values were measured (0.05 ± 0.01 for BS-1 and BS-2), remarkably higher values were found in the case of the materials obtained as powders [0.38 ± 0.04 (BS-3) and 0.33 ± 0.03 (BS-4)]. We emphasize that the quantum yield values found for BS-3 and BS-4 hybrids are the largest found for amine-functionalized siloxane-based hybrids lacking aromatic groups. Larger values (0.43 ± 0.04) were only found for bipyridine-based BS hybrids (Graffion et al., 2012) due to the contribution of the bipyridine triplet states that add an extra emission component to the amine- and siliceous-related contributions. It is of interest to mention at this stage that the maximum quantum yield measured by Green et al. (1997) for a series of highly emissive broadband phosphors synthesized from a tetraalkoxysilane sol-gel precursor and a variety of organic carboxylic acids and thus lacking metal activator ions was found in the case of the 3-aminopropyltriethoxysilane (APTES)–formic acid material with an external quantum yield of 0.35 ± 0.10 at a 365-nm excitation wavelength, a value practically identical to that exhibited by BS-3.

It is not casual that the synthesis of BS-3, the material that exhibits the highest quantum yield value, involved the addition of HCl. The major role exerted by an acid catalyst in the increase of emission quantum yield values was reported for mono/di-amidosil hybrids (Nunes et al., 2012, 2015b) and tripodal poly(oxypropylene) (POP)-siloxane hybrids (tri-ureasils) (Freitas et al., 2012). Moreover, it was demonstrated that the incorporation of an acid or a basic catalyst in the synthesis procedure is of prime importance for the development of materials comprising a hydrogen-bonded amide–amide array whose degree of order falls into the optimal range that yields the highest emission quantum yield values (Nunes et al., 2015b).

We will now try to explain the reason for the dramatic difference observed between the emission quantum yields of BS-1/BS-2 and BS-3/BS-4. The emergence of two distinct morphologies in the four BSs was detailed above (Figure 8). We recall that in the case of BS-1 and BS-2 a very slow hydrolysis led to the formation of lamellar ribbons A. These then yielded 3D thin platelets comprising ordered amine–amine hydrogen bonding interactions preventing efficient electron–hole recombination processes in the amine groups. In the case of BS-3 and BS-4 the very fast hydrolysis resulted in the growth of thin, bent ribbons and ultimately to 3D onion-like nanoparticles, including amine-amine hydrogen bonded aggregates with a much lower degree of order, facilitating radiative recombination processes. In addition, in the latter two BSs, because of the presence of HCl and NaOH in the reaction media, a fraction of the = NH groups was protonated (= ${\text{NH}}_{2}^{+}$-) and deprotonated (= N^−^), respectively. The short distance between = NH/ = ${\text{NH}}_{2}^{+}$ and = NH/ = N^−^ pairs, respectively, allowed fast proton hopping probably via a classical Grotthus mechanism. This induced us to suppose that BS-3 and BS-4 might be seen as proton conducting and proton-vacancy conducting hybrids, respectively. In spite of the low concentration of acid and base present in these two samples, a non-negligible ionic conductivity was measured for BS-3 and BS-4 (2.5 × 10^−11^ and 1.3 × 10^−11^ S cm^−1^, respectively, Supplementary Figure 11). Interestingly, the trend observed matches perfectly that of the emission quantum yields. These findings beautifully confirm our claims.

## Conclusion

Four amine-functionalized BSs were produced by the sol-gel process and self-directed assembly from BTMSPA through a synthetic route in which the nature/concentration of the solvents, and the presence (or not)/type of catalyst, exerted a major influence in terms of structure, morphology, wettability and optical properties. In these materials face-to-face assembly of flat lamellae (large excess of THF and no catalyst, BS-1/BS-2) or folded lamellae (large excess of water only or water/ethanol and catalyst, BS-3/BS-4) led to the formation of platelets or onion-like nanoparticles, and ultimately to transparent films or powders, respectively. The comprehensive mechanistic scenario proposed for the formation of the hybrids is based on two major premises: (1) The unique sol-gel reactivity of BTMSPA; (2) The key role of the synthesis solvents as homogenizing agents.

The present work, in which BTMSPA was revisited, opens exciting prospects for the rational solvent-mediated design of multifunctional amine-rich ordered BSs. The BS-1 and BS-2 hybrids are extremely attractive because they were easily processed as quasi-hydrophobic free-standing transparent films, thus lending themselves to application in a wide variety of areas as coatings. The BS-3 and BS-4 materials have a bright future in the field of optics and solid state electrochemistry. In fact, their synthesis via an acid- or base-catalyzed hydrolytic route, respectively, boosted the emission quantum yield to values that are the largest ever reported for amine-functionalized siloxane-based hybrids lacking aromatic groups. In these two BSs the onion-like morphology comprises hydrogen bonded aggregates with a low degree of order which facilitates radiative recombination processes associated with fast Grotthus proton hopping between = ${\text{NH}}_{2}^{+}$/ = NH groups and = N^−^/ = NH groups, promoted by H^+^ and OH^−^ ions, respectively, and assisted by the short amine-amine distance. The non-negligible proton conductivity displayed by BS-3 and BS-4 justifies further studies of these materials targeting their use as proton or proton-vacancy conducting electrolytes, respectively, for fuel cells.

The co-condensation of BTMSPA with other silica sources, such as tetraethylortosilicate (TEOS) or tetramethylortosilicate (TMOS), the use of other precursor molecules bearing more than one amine group, and the introduction of longer alkyl chains are being considered currently with the goal of creating more robust frameworks with significantly higher degree of order, while simultaneously enhancing the emission quantum yield values and the protonic conductivity. In addition, solvents with other physical-chemical properties are being employed.

## Materials and methods

### Chemicals

Bis(3-trimethoxysilylpropyl)amine (BTMSPA, 96%, Gelest Inc.) was used as received. Ethanol (CH_3_CH_2_OH, 99.9%, Merck), tetrahydrofuran (THF, 99.9%, Merck), and acetone (CH_3_COCH_3_, 99.9%, Fluka) were stored over molecular sieves. Hydrochloric acid (HCl, 37%) and sodium hydroxide (NaOH, ≥ 98%) were purchased from Sigma-Aldrich. High-purity deionized water was used in all the experiments.

### Synthesis

Four BSs hybrid samples were synthesized according to the procedures described as follows (Moreau et al., 2001; Nunes et al., 2015b) and summarized in Supplementary Figure 1. Relevant details of the synthesis are given in Supplementary Table 1.

#### Sub-stoichiometric/stoichiometric water content and large amount of THF

A mixture of ethanol and water was added to a THF solution of BTMSPA to promote the hydrolysis and condensation reactions characteristic of the sol-gel process. The resulting mixture (pH = 8) was stirred at room temperature in a sealed flask for 90 min and then cast onto a Teflon mold, which was covered with Parafilm and left in a fume cupboard for 48 h. The mold was subsequently transferred to an oven at 50°C and the sample was aged for a period of 2 weeks. These BSs were labeled as BS-1 (molar ratio BTMSPA:H_2_O = 1:1.5) and BS-2 (molar ratio BTMSPA:H_2_O = 1:3).

#### Hyper-stoichiometric water content and large amount of THF

A large amount of water and HCl were added to a THF solution of BTMSPA (pH = 9). When water was added a white precipitate was immediately formed. The mixture was stirred at room temperature during 1 week. The solid was filtered and washed with water until neutral pH was attained and was again washed with ethanol and acetone. The solid powder was then dried at 50°C during 1 week. This BS was represented by the notation BS-3.

#### Hyper-stoichiometric water content, large amount of ethanol and base catalysis

The BTMSPA precursor was dissolved in ethanol. Water was then added yielding a white suspension. Its pH was adjusted to 12 through the addition of an aqueous solution of NaOH. This solution was heated to 60°C under static conditions for 1 week. After a few days in static conditions a white crystalline solid was formed. After filtration, the solid powder was washed successively with water, ethanol and acetone. This BS was identified with the notation BS-4.

### Characterization

^29^Si Magic Angle Spinning (MAS) and ^13^C Cross Polarization (CP)/MAS NMR spectra were recorded on a Bruker Avance 400 (9.4 T) spectrometer at 79.49 and 100.62 MHz, respectively. ^13^C CP/MAS NMR spectra were recorded with 4 μs 1H 90° pulse, 2 ms contact time, a recycle delay of 4 s and at a spinning rate of 8 kHz. ^29^Si MAS NMR spectra were recorded with 2 μs (~30°) rf pulses, and recycle delay of 60 s and at a 5.0 kHz spinning rate. The chemical shifts (δ) are quoted in parts per million relative to tetramethylsilane (Me_4_Si).

The small and wide angle X-ray scattering (SWAXS) experiments were conducted using a Guinier-Mering setup with a 2D image plate detector. The X-ray source was a molybdenum anode, which delivered a high-energy monochromatic beam (λ = 0.71 Å, E = 17.4 keV), providing structural information over scattering vectors *q* ranging from 0.04 to 25 nm^−1^. Helium flowed between the sample and the image plate to avoid air adsorption. The sample acquisition time was 3600 s and the glass capillaries (Higenberg) had a thickness of 2 mm. The image azimuthal average was determined by the FIT2D software from ESRF (France) and data corrections, radial averaging were performed *via* standard procedures.

Nitrogen (N_2_) adsorption–desorption isotherms were performed with a Micromeritics Gemini 2375 system at −196°C. The material was degassed overnight at 200°C prior to being measured. The surface area measurements were carried out according to the Brunauer–Emmett–Teller (BET) method.

HR-SEM micrographs of the samples produced were obtained using a FEI Quanta 400FEG/Edax Genesis X4M scanning electron microscope at CEMUP-Porto (CEMUP-Porto contracts REEQ/1062/CTM/2005 and REDE/1512/RME/2005 FCT). Images were obtained at high or low vacuum, depending on the sample charging effects, and in the secondary electrons or backscattered electrons modes. Prior to being analyzed the samples were coated with gold (Au)/palladium (Pd).

SEM images were obtained at 20 kV on a Hitachi S-3400N type II microscope equipped with a Bruker x-flash 5010 at high vacuum. The sample was coated with Au.

TEM micrographs were obtained using a Hitachi H9000na microscope operating at 300 kV. The samples for TEM analysis were previously dispersed in ethanol. A drop of this suspension was then poured onto a 400 mesh copper grid coated with a carbon film and the solvent allowed evaporating at room temperature.

The DSC curves of the samples were recorded at a heating rate of 10°C min^−1^ under a flowing argon atmosphere. Each sample was placed in a 40 μL perforated aluminum pan and the thermogram was recorded using a Mettler DSC 821e. The samples were heated from 0 to 175°C, then cooled down to 0°C and then re-heated up to 300°C.

Thermogravimetric analysis (TGA) was performed on a Netzsch TG 209F3 equipment and using alumina (Al_2_O_3_) crucibles. The measurements were conducted between 25 and 800°C, at a heating rate of 10°C min^−1^, and under a nitrogen atmosphere with 50 mL min^−1^ rate flow.

ATR/FT-IR spectra were registered in a Thermo Scientific Nicolet iS50 FTIR spectrometer, resorting to an ATR accessory with a diamond crystal. The instrument is controlled by the Omnic software package (version 9.2.28) from Thermo Fisher Scientific Inc. The spectra were collected in the 4,000–400 cm^−1^ range by averaging 64 scans at a resolution of 4 cm^−1^.

The surface wettability of the samples was assessed by means of static contact angle measurements using the sessile drop method. Contact angles (θ) were measured at room temperature with ultra-pure distilled water using a Contact Angle OCAH 200 (DataPhysics Instruments) and SCRA-20 software. The samples were analyzed as pellets. The volume of the liquid droplets was kept constant at 2 μL. The θ values were measured at three different spots and the results reported correspond to the average value of the three measurements. The error analysis of the data was implemented by arithmetic mean of the root mean square error.

The photoluminescence spectra were recorded at 300 K with a modular double grating excitation spectrofluorimeter with a TRIAX 320 emission monochromator (Fluorolog-3, Horiba Scientific) coupled to a R928 Hamamatsu photomultiplier, using a front face acquisition mode. The excitation source was a 450 W Xe arc lamp. The emission spectra were corrected for detection and optical spectral response of the spectrofluorimeter and the excitation spectra were corrected for the spectral distribution of the lamp intensity using a photodiode reference detector. The room temperature emission quantum yields were measured using the Hamamatsu C9920-02 setup with a 150 W Xe lamp coupled to a monochromator for wavelength discrimination, an integration sphere as sample chamber and a multichannel analyzer for signal detection. Three measurements were made for each sample and the average values obtained are reported with accuracy within 10% according to the manufacturer.

The electrical resistivity of the powder samples (BS-3 and BS-4) was obtained by measuring the characteristic I–V curves at room temperature with a Keithley 6487 picoammeter/voltage source. BS-3 and BS-4 hybrids were finely ground and pressed into pellets with a thickness of 117 ± 3 μm and 108 ± 4 μm, respectively. The samples were located between aluminum electrodes and placed on the sample holder. The current and voltage (−1.0 to 1.0 V) were measured and the electrical resistivity (ρ) was calculated using the equation R = ρ l/A, where *R* is the electrical resistance, l the thickness and *A* the area of the sample.

## Author contributions

RP: synthesized the samples, registered the DSC and I–V curves, organized all data, made the general scheme, was deeply involved in the discussion of the characterization data and wrote the first draft of the manuscript; SN: recorded the static contact angle data and SEM images; GT: recorded the SWAXS spectra; MC: obtained the POM images; AV: registered the TGA curves; MF: recorded the TEM images; MS: contributed to the discussion of the thermal data; LC and RF: recorded and discussed the photoluminescence data; VdZB: proposed the study and was deeply involved in the discussion of the results and in the writing of the manuscript. All authors contributed to manuscript revision, read and approved the submitted version.

### Conflict of interest statement

The authors declare that the research was conducted in the absence of any commercial or financial relationships that could be construed as a potential conflict of interest.
