# Supplementary material for: Novel Highly Luminescent Amine-Functionalized Bridged Silsesquioxanes
Source: Front Chem. 2018 Jan 15;5:131. doi: 10.3389/fchem.2017.00131 (PMC5775297; doi:10.3389/fchem.2017.00131)
Supplement: Supplementary file 1 [file Table1.PDF]

Supplementary Material

Novel highly luminescent amine-functionalized bridged silsesquioxanes

Rui F. P. Pereira,[1*] Sílvia C. Nunes,[2] Guillaume Toquer,[3] Marita A. Cardoso,[4] Artur J.M. Valente,[5] Marta C. Ferro,[6] Maria M. Silva,[1] Luís D. Carlos,[7] Rute A. S. Ferreira,[7] Verónica de Zea Bermudez[4*]

*** Correspondence:** Rui F.P. Pereira: rpereira@quimica.uminho.pt; Verónica de Zea Bermudez: vbermude@utad.pt

Supplementary Table 1. Relevant details of the synthesis of the BSs hybrids.

Sample	BTMSPA		H_2O		CH_3CH_2OH		THF		pH	Molar Proportion
	m *(g)*	*n* *(mmol)*	*V* *(µL)*	*n* *(mmol)*	*V* *(µL)*	*n* *(mmol)*	*V* *(mL)*	*n* *(mmol)*		*BTMSPA:H₂O:CH₃CH₂OH:THF*
**BS-1**	1.00	2.93	79.1	4.39	684	11.7	20.0	244.1	8	1 : 1.5 : 4 : 83.4
**BS-2**	1.01	2.96	160	8.87	1381	23.7	20.0	244.1		1 : 3 : 8 : 82.5
**BS-3**					**HCl 1.0 M**					*BTMSPA:H_2O:HCl:THF*
					V *(µL)*	*n* *(mmol)*				
	1.02	2.99	32.4	1.79	598	0.60	17.1	209.3	9	1 : 600 : 0.2 : 70
**BS-4**					**CH_3CH_2OH**		**NaOH 2.0 M**			*BTMSPA:H_2O:CH_3CH_2OH:NaOH*
					V *(mL)*	*n* *(mmol)*	*V* *(µL)*	*n* *(mmol)*		
	1.02	2.99	17.1	940	6.80	116.8	344	0.69	12	1 : 315 : 39 : 0.23
