# Supplementary material for: Novel Highly Luminescent Amine-Functionalized Bridged Silsesquioxanes
Source: Front Chem. 2018 Jan 15;5:131. doi: 10.3389/fchem.2017.00131 (PMC5775297; doi:10.3389/fchem.2017.00131)
Supplement: Supplementary file 2 [file Table2.PDF]

Supplementary Material

Novel highly luminescent amine-functionalized bridged silsesquioxanes

Rui F. P. Pereira,[1*] Sílvia C. Nunes,[2] Guillaume Toquer,[3] Marita A. Cardoso,[4] Artur J.M. Valente,[5] Marta C. Ferro,[6] Maria M. Silva,[1] Luís D. Carlos,[7] Rute A. S. Ferreira,[7] Verónica de Zea Bermudez[4*]

*** Correspondence:** Rui F.P. Pereira: rpereira@quimica.uminho.pt; Verónica de Zea Bermudez: vbermude@utad.pt

Supplementary Table 2. Time dependence of the average contact angle (θ) values for the BS hybrids.

	θ (°)					
Sample	t = 0 s	t = 0.1 s	t = 1 s	t = 3 s	t = 5 s	t = 10 s
BS-1	-	90.1 ± 3.0	-	-	86.6 ± 3.0	86.3 ± 3.0
BS-2	77.8 ± 0.7	77.3 ± 1.7	77.2 ± 2.9	75.4 ± 1.8	75.4 ± 1.8	75.1 ± 1.2
BS-3	88.7 ± 4.1	75.0 ± 2.8	65.0 ± 3.0	57.6 ± 6.6	53.4 ± 7.4	42.2 ± 12.2
BS-4	68.0 ± 4.2	57.3 ± 1.1	45.0 ± 3.2	28.6 ± 0.6	16.6 ± 2.3	-
