# Supplementary material for: Novel Highly Luminescent Amine-Functionalized Bridged Silsesquioxanes
Source: Front Chem. 2018 Jan 15;5:131. doi: 10.3389/fchem.2017.00131 (PMC5775297; doi:10.3389/fchem.2017.00131)
Supplement: Supplementary file 6 [file Image4.PDF]

Supplementary Material

Novel highly luminescent amine-functionalized bridged silsesquioxanes

Rui F. P. Pereira,[1*] Sílvia C. Nunes,[2] Guillaume Toquer,[3] Marita A. Cardoso,[4] Artur J.M. Valente,[5] Marta C. Ferro,[6] Maria M. Silva,[1] Luís D. Carlos,[7] Rute A. S. Ferreira,[7] Verónica de Zea Bermudez[4*]

*** Correspondence:** Rui F.P. Pereira: rpereira@quimica.uminho.pt; Verónica de Zea Bermudez: vbermude@utad.pt

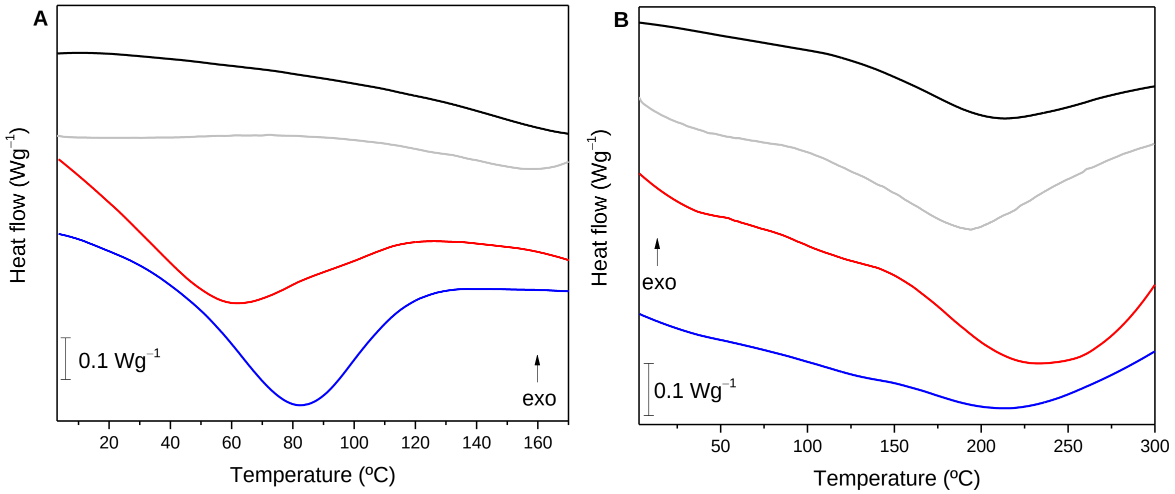

Supplementary Figure 4. DSC thermograms of the BS-1 (black line), BS-2 (grey line), BS-3 (red line) and BS-4 (blue line) hybrids under two successive heating cycles: (A) 0 to 170 °C (1st cycle) and (B) 0 to 300 °C (2nd cycle).

Supplementary Figure 4 shows the DSC thermograms of BS-1, BS-2, BS-3 and BS-4 BSs under two successive heating cycles. The first heating cycle (A) (from 0 to 170 °C) revealed a broad endothermic event in the case of BS-3 (onset temperature (T_{onset}) = 52 °C, endset temperature (T_{endset})= 108 °C and enthalpy (ΔH) = −101 J g^{-1}) and BS-4 (T_{onset} = 38 °C, T_{endset} = 119 °C and ΔH = 113 J g^{-1}) hybrids. This endothermic event is associated with the presence of adsorbed solvents (water/ethanol). For BS-2 this thermal event is only slightly denoted (T_{onset} = 120 °C, T_{endset} = 170 °C and ΔH = −7 J g^{-1}). BS-1 did not produce this thermal event, but the profile of the curve suggests the occurrence of an endothermic event peaking at a temperature higher than 160 °C. In the DSC curves corresponding to the second heating cycle (B) an endothermic broad peak centered at 210, 192, 240 and 210 °C is seen in the DSC curves of BS-1, BS-2, BS-3 and BS-4, respectively.
