# Supplementary material for: Novel Highly Luminescent Amine-Functionalized Bridged Silsesquioxanes
Source: Front Chem. 2018 Jan 15;5:131. doi: 10.3389/fchem.2017.00131 (PMC5775297; doi:10.3389/fchem.2017.00131)
Supplement: Supplementary file 9 [file Image7.PDF]

Supplementary Material

Novel highly luminescent amine-functionalized bridged silsesquioxanes

Rui F. P. Pereira,[1*] Sílvia C. Nunes,[2] Guillaume Toquer,[3] Marita A. Cardoso,[4] Artur J.M. Valente,[5] Marta C. Ferro,[6] Maria M. Silva,[1] Luís D. Carlos,[7] Rute A. S. Ferreira,[7] Verónica de Zea Bermudez[4*]

*** Correspondence:** Rui F.P. Pereira: rpereira@quimica.uminho.pt; Verónica de Zea Bermudez: vbermude@utad.pt

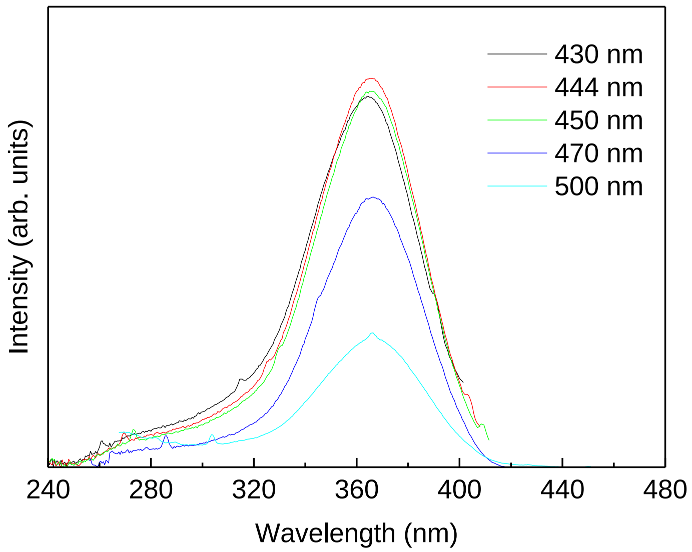

Supplementary Figure 7. Excitation spectra of BS-3 monitored at different wavelengths.

of the BS-3 (red) and BS-4 (blue) hybrids.
