# Supplementary material for: Novel Highly Luminescent Amine-Functionalized Bridged Silsesquioxanes
Source: Front Chem. 2018 Jan 15;5:131. doi: 10.3389/fchem.2017.00131 (PMC5775297; doi:10.3389/fchem.2017.00131)
Supplement: Supplementary file 10 [file Image8.PDF]

*Supplementary Material*

# Novel highly luminescent amine-functionalized bridged silsesquioxanes

**Rui F. P. Pereira,[1*] Sílvia C. Nunes,[2] Guillaume Toquer,[3] Marita A. Cardoso,[4] Artur J.M. Valente,[5] Marta C. Ferro,[6] Maria M. Silva,[1] Luís D. Carlos,[7] Rute A. S. Ferreira,[7] Verónica de Zea Bermudez[4*]**

*** Correspondence:** Rui F.P. Pereira: rpereira@quimica.uminho.pt; Verónica de Zea Bermudez: vbermude@utad.pt

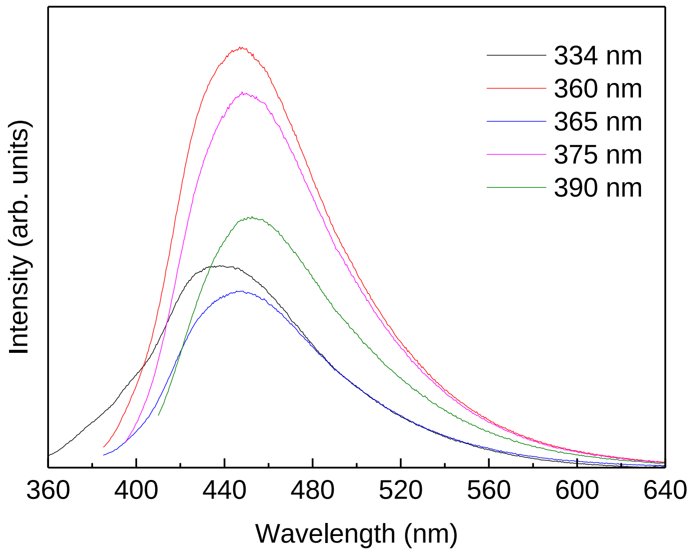


**Supplementary Figure 8.** Emission spectra of BS-1 excited at different wavelengths.
